# A novel sulfur dioxide probe inhibits high glucose-induced endothelial cell senescence

**DOI:** 10.3389/fphys.2022.979986

**Published:** 2022-12-16

**Authors:** Hui Ren, WenWen Han, Shuo Wang, BaoXiang Zhao, JunYing Miao, ZhaoMin Lin

**Affiliations:** ^1^ Shandong Provincial Key Laboratory of Animal Cells and Developmental Biology, School of Life Science, Shandong University, Qingdao, China; ^2^ Institute of Organic Chemistry, School of Chemistry and Chemical Engineering, Shandong University, Jinan, China; ^3^ Institute of Medical Science, The Second Hospital of Shandong University, Jinan, China

**Keywords:** sulfur dioxide, high glucose, vascular endothelial cell senescence, lipid droplets, lysosomes, LAMP1

## Abstract

Sulfur dioxide (SO_2_) is an important gas signal molecule produced in the cardiovascular system, so it has an important regulatory effect on human umbilical vascular endothelial cells (HUVECs). Studies have shown that high glucose (HG) has become the main cause of endothelial dysfunction and aging. However, the mechanism by which SO_2_ regulates the senescence of vascular endothelial cells induced by HG has not yet been clarified, so it is necessary to find effective tools to elucidate the effect of SO_2_ on senescence of HUVECs. In this paper, we identified a novel sulfur dioxide probe (2-(4-(dimethylamino)styryl)-1,1,3-trimethyl-1H-benzo [e]indol-3-ium, DLC) that inhibited the senescence of HUVECs. Our results suggested that DLC facilitated lipid droplets (LDs) translocation to lysosomes and triggered upregulation of LAMP1 protein levels by targeting LDs. Further study elucidated that DLC inhibited HG-induced HUVECs senescence by promoting the decomposition of LDs and protecting the proton channel of V-ATPase on lysosomes. In conclusion, our study revealed the regulatory effect of lipid droplet-targeted sulfur dioxide probes DLC on HG-induced HUVECs senescence. At the same time, it provided the new experimental evidence for elucidating the regulatory mechanism of intracellular gas signaling molecule sulfur dioxide on vascular endothelial fate.

## 1 Introduction

In the past, sulfur dioxide (SO_2_) was regarded as a waste gas in air pollution. However, biochemical studies on amino acid metabolism have shown that SO_2_ can be endogenously generated in cardiovascular tissues (Du et al., 2008). As a novel gas signaling molecule, mounting evidence has shown that SO_2_ can alleviate a variety of cardiovascular diseases, such as hypertension, atherosclerosis, vascular calcification, aging endothelial dysfunction, myocardial injury, hypoxic pulmonary hypertension (Huang et al., 2022). However, whether and how HUVECs-derived endogenous SO_2_ affects cell senescence under physiological and pathophysiological conditions remain largely unexplored.

A high concentration of glucose in the blood is harmful to vascular endothelial cells (Eelen et al., 2015). Numerous studies demonstrate that culturing cells in high concentration of glucose accelerates cellular senescence (Wiley and Campisi, 2021). For instance, HG accelerates endothelial cell senescence, apoptosis and autophagy by inducing renin-angiotensin system-mitochondrial damage (Chen et al., 2014). In addition, vascular endothelial cells are the main site of sulfur dioxide production. Consequently, exploring novel sulfur dioxide regulatory factors to inhibit senescence caused by hyperglycemia on endothelial cells is of great significance.

The presence of lysosome is important to cellular senescence. Lysosomes consist of an acidic lumen and a monolayer of lysosomal membranes (Zhang et al., 2021). Lysosomal membranes contain hundreds of membrane proteins, among which lysosome-associated membrane proteins (LAMP1 and LAMP2) have been shown to be involved in processes such as autophagy, lipid transport and aging (Alessandrini et al., 2017). Lysosomes maintain an acidic lumen by means of the proton pump and the vacuolar H-ATPase (V-ATPase) (Wang et al., 2014). V-ATPases are ATP-driven proton pumps that transport protons across lysosomal membranes to activate the activities of various intracellular hydrolases and acidify intracellular compartments (Forgac, 2007). Increasing attention has been focused on the connection between cellular senescence and lysosomal acidification as substantial evidence suggests that v-ATPase activity is altered and lysosomal pH is dysregulated in the process of cellular aging (Wang et al., 2018). Lysosomal acidification disorder increases the risk of cellular senescence and death. Studies have shown that V-ATPase-mediated acidification of vacuoles (equivalent to lysosomes in mammals) has been identified as a positive regulator of lifespan in yeast (Hughes and Gottschling, 2012). Therefore, targeting V-ATPase to modulate lysosome acidity is a promising pathway to inhibit aging-related diseases.

In a previous work we synthesized and identified a novel ratiometric fluorescent probe 2-(4-(dimethylamino)styryl)-1,1,3-trimethyl-1H-benzo [e]indol-3-ium (DLC) for sensitively and selectively detecting endogenous sulfur dioxide in living cells with fast response (Yan et al., 2020). The activity of lysosomes and lysosome acidity played a crucial role in senescence, but it is unclear whether DLC regulates HUVECs fate by affecting lysosomes. Therefore, in this article we investigated the effects of DLC on HUVECs senescence as well as the underlying mechanism.

## 2 Materials and methods

### 2.1 Chemicals

DLC was synthesized as described and dissolved in DMSO (Sigma-Aldrich, D2650) to make a 0.1 M stock solution (Yan et al., 2020). MAFP (MCE, HY-103334) and CQ (MCE, HY-17589A) were purchased from MCE.

### 2.2 Cell culture

Source of HUVECs from human umbilical vein was performed as described previously (Zhao et al., 2020). HUVECs were cultured in M199 medium (Gibco, Grand Island, NY) supplemented with 15% fetal bovine serum (FBS, Hyclone Lab, Tauranga, New Zealand) and 2 ng/ml FGF-2 at 37°C under humidified conditions and 5% CO_2_. All HUVECs involved in experiments were at no more than passage 30. We used a high concentration glucose (30 mM glucose, Sigma-Aldrich, G8769) to induce senescent cells. We treated HUVECs with normal concentration glucose (5.5 mM glucose) (NG), high concentration glucose (30 mM glucose), and HG + DLC in all subsequent experiments.

### 2.3 Senescence-associated β-galactosidase assay

HUVECs were exposed to 4% tissue cell fixative solution for fixation. After 15 min, 1 × PBS washed three times and warmed Then, cells were incubated with SA-β-gal staining solution (Genview, LX369) for more than 18 h at 37°C. Stain of senescent cells was observed blue under an inverted phase-contrast microscope. The percentage of positive cells was estimated by counting at least 150 cells in each well.

### 2.4 Staining and identification of lipid droplets

Lipid droplets assay was consistent with the previous description (Ren et al., 2021). After treatment, HUVECs were incubated with lipid droplet dye (1:300 dilution in 0.1 M PBS) HCS Lipid TOX™ Deep Red neutral lipid stain (Invitrogen, M22426) for 30 min. Lipid droplets were observed by laser scanning confocal microscopy (Zeiss LSM900, Carl Zeiss Canada) The relative number of LDs was quantified by ImageJ software.

### 2.5 Western blot analysis

HUVECs were lysed in western and IP buffer (Beyotime, P0013) containing proteinase inhibitor PMSF (Sigma-Aldrich). After centrifuging at 12,000 × g, 4°C for 15 min, the supernatant was collected. Protein samples were loaded on 12% or 9% SDS/PAGE at 4°C and then electrophoretically transferred to polyvinylidene difluoride (PVDF) membrane (Millipore, IPVH00010). The membranes were blocked with 5% nonfat milk for 1 h at room temperature, incubated with primary antibodies: LAMP1(Cell Signaling Technology, 9091); P21(Cell Signaling Technology, 2947); PLIN2 (Abcam, ab-108323); ACTB (Sigma-Aldrich, 122M4782); GAPDH (Santa Cruz Biotechnology sc-47724) at 4°C overnight, then horseradish peroxidase-linked secondary antibodies (Jackson ImmunoResearch, goat anti-rabbit:13963, goat anti-mouse:130389). Fluorescence signals were detected with X-ray films after being incubated with Immobilon Western Chemiluminescent HRP substrate for 3 min. The relative quantity of proteins was quantified by ImageJ software.

### 2.6 Immunofluorescence microscopy

HUVECs were fixed in 4% paraformaldehyde for 15 min. After washing three times with 1 × PBS cells were permeated with 0.1%–0.2% TritonX-100 for 5 min, and then blocked with 5% donkey serum for 30 min. Then cells were incubated with primary antibodies overnight at 4°C and then incubated cells with corresponding secondary antibodies for 60 min at 37°C. Fluorescence intensity was detected by laser scanning confocal microscopy (Zeiss LSM900, Carl Zeiss Canada).

### 2.7 Lysosomal pH sensing experiment

After treatment, HUVECs were grown on confocal dishes and then cells were treated with 0.5 μM Lysosensor™ Green DND-189 (pH = 5.2) for 30 min. After washing cells with 1 × PBS three times, fluorescence was detected by a confocal laser scanning (Carl Zeiss, Germany). The excitation wavelength of Lysosensor™ Green DND-189 is 443 nm.

### 2.8 RNA interference

The specific siRNA against LAMP1and scramble siRNA were purchased from Tsingke (Tsingke, China). Cells at 70%–80% confluence were transfected with siRNA against LAMP1 and scramble siRNA by use of Lipofectamin 2000 (Invitrogen, 11668–019) transfection reagent according to the manufacturer’s instructions.

### 2.9 Statistical analyses

Experimental results were analyzed by one-way ANOVA and reported as mean ± SEM. Graph Pad Prism software (version 5.0) was used for statistical analysis. All experiments were repeated at least three times independently. *p* < 0.05 was regarded as statistically significant.

## 3 Results

### 3.1 DLC co-localized with lipid droplets

**FIGURE 1:**
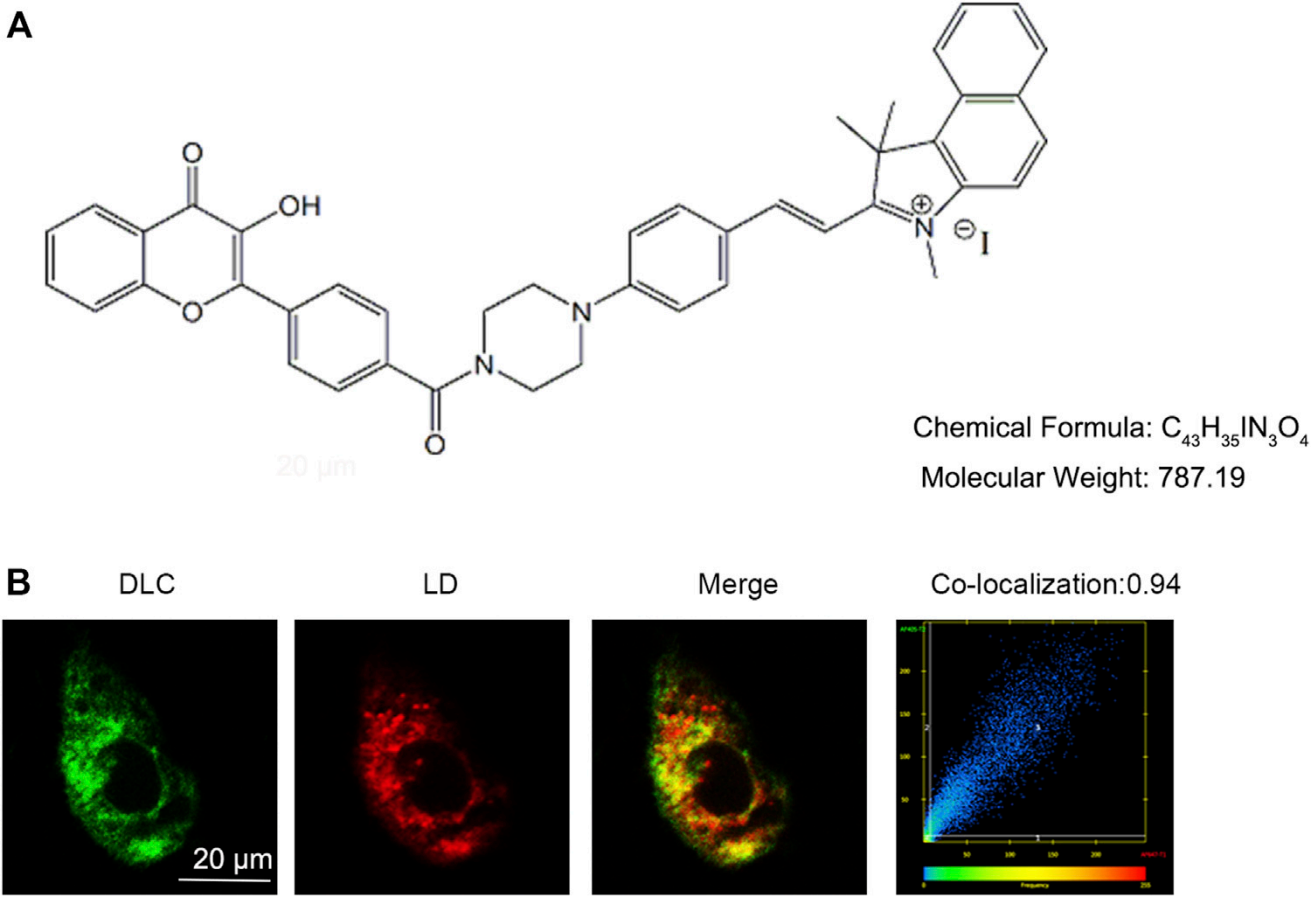
DLC co-localized with lipid droplets. **(A)** The chemical structure of DLC. **(B)** HUVECs were treated with 1 μM DLC for 12 h and stained with HCS Lipid TOX™ Deep Red neutral lipid stain. Co-localization coefficient of DLC and LDs was 0.94 in HUVECs. Scale bar, 20 μm.

Small chemical probe targeting SO_2_ in different organelles had varied effects on cell fate. The chemical structure of the probe DLC is shown in Figure 1A. To explore the distribution of DLC in the organelles, we investigated the organelle targeting of DLC based on its good fluorescence properties. The results revealed that DLC co-localized with LDs in HUVECs (Figure 1B).

### 3.2 DLC inhibited HG-induced senescence of HUVECs

**FIGURE 2:**
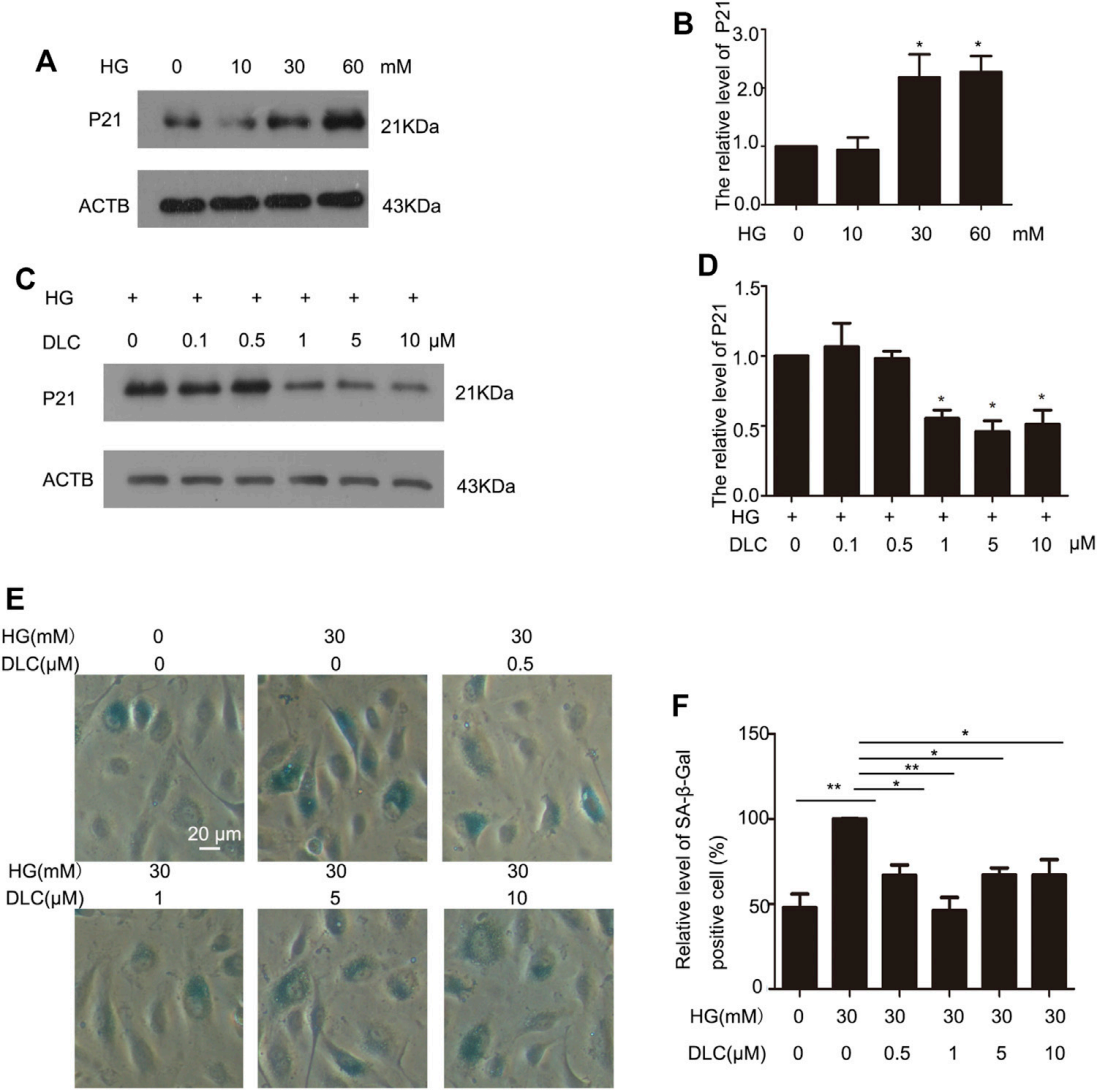
DLC inhibited HG-induced senescence of HUVECs. **(A,B)**. HUVECs were treated with HG (0, 10, 30, 60 mM) for 24 h and protein levels of p21 were determined by western blot. **(C,D)**. HUVECs were treated with DLC (0, 0.5, 1, 5, 10 μM) for 24 h under HG (30 mM) condition and protein levels of p21 were determined by Western blot. **(E,F)**. HUVECs were treated with DLC (0,0.5,1,5,10 μM) for 24 h under HG (30 mM) condition, cells were detected SA-β-Gal staining. *p < 0.05, **p < 0.01, *n* = 3.

Cell cycle protein dependent kinase inhibitor P21 is an important marker of cell senescence. We treated the cells with various concentrations of glucose (0, 10, 30, and 60 mM) for 24 h to investigate the effects of HG on senescence. The results revealed that P21was remarkably elevated, which suggested that HG can induce senescence of HUVECs (Figures 2A, B). Furthermore, we found that DLC treatment significantly decreased the protein level of p21 in HG-induced senescence of HUVECs (Figures 2C, D). Then we examined the effect of DLC on senescence using SA- β-gal staining. We observed there were more positively stained cells in the HG group than in the control group (Figures 2E, F). Meanwhile, we found that DLC significantly reduced the percentage of SA-β-gal positive cells. These data demonstrated that DLC treatment significantly suppressed the senescent biomarkers in HUVECs, DLC could inhibited HG-induced senescence of HUVECs.

### 3.3 DLC suppressed lipid droplet number

**FIGURE 3:**
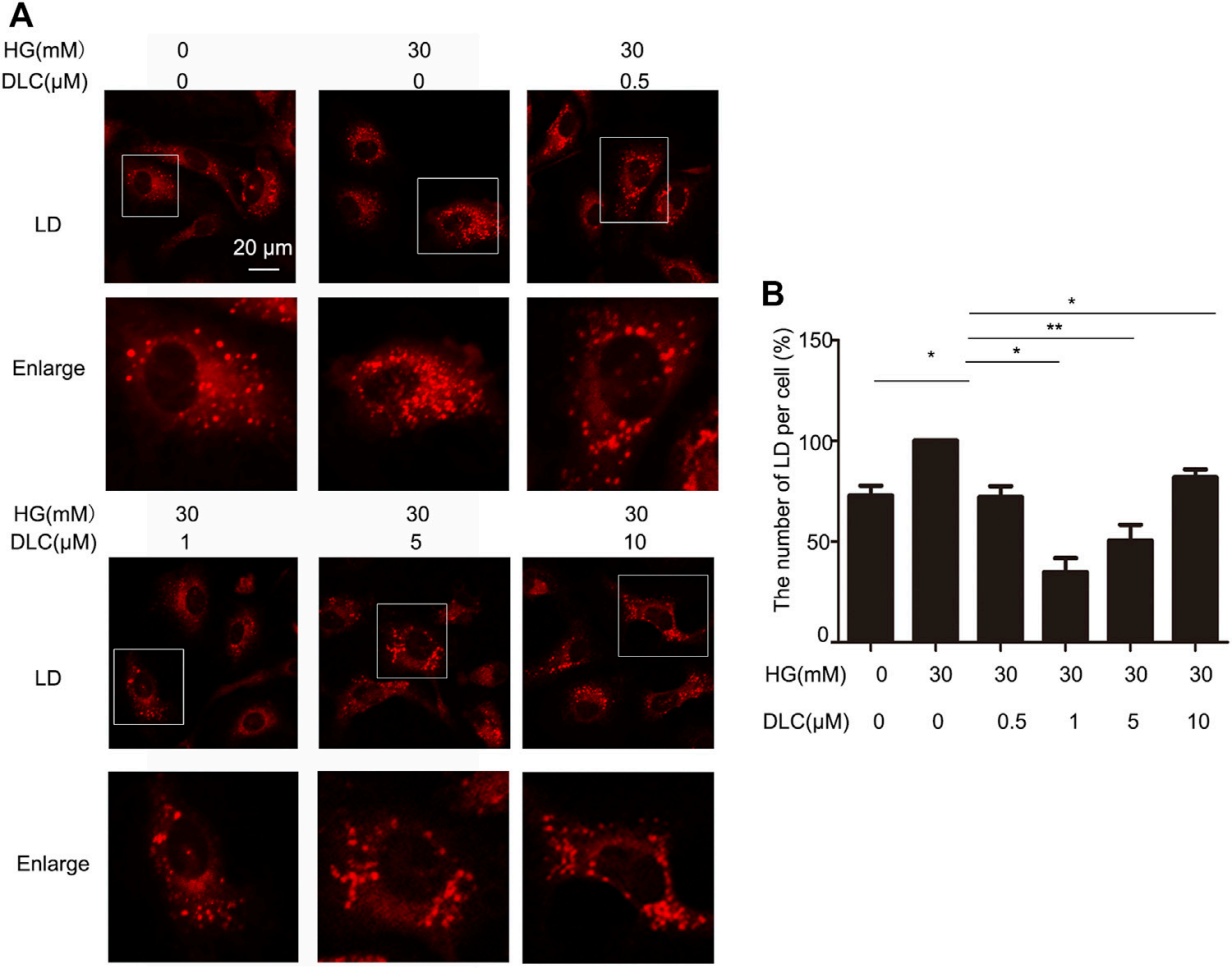
DLC suppressed lipid droplet number in HUVECs. **(A,B)** HUVECs were treated with DLC (0,0.5,1,5,10 μM) for 24 h under HG (30 mM) condition and stained with neutral lipid stain. The number of LDs was counted by ImageJ. Scale bar, 20 μm *p < 0.05, **p < 0.01, *n* = 3.

In order to explore the relation between DLC and LDs, we treated HUVECs with DLC and stained with LD tracker (HCS Lipid TOX™ Deep Red neutral lipid stain). We found that DLC suppressed lipid droplet number (Figure 3). The results showed that HG significantly increased the number of LDs in HUVECs, while DLC decreased the number of LDs under HG condition.

### 3.4 DLC promoted degradation of LDs

**FIGURE 4:**
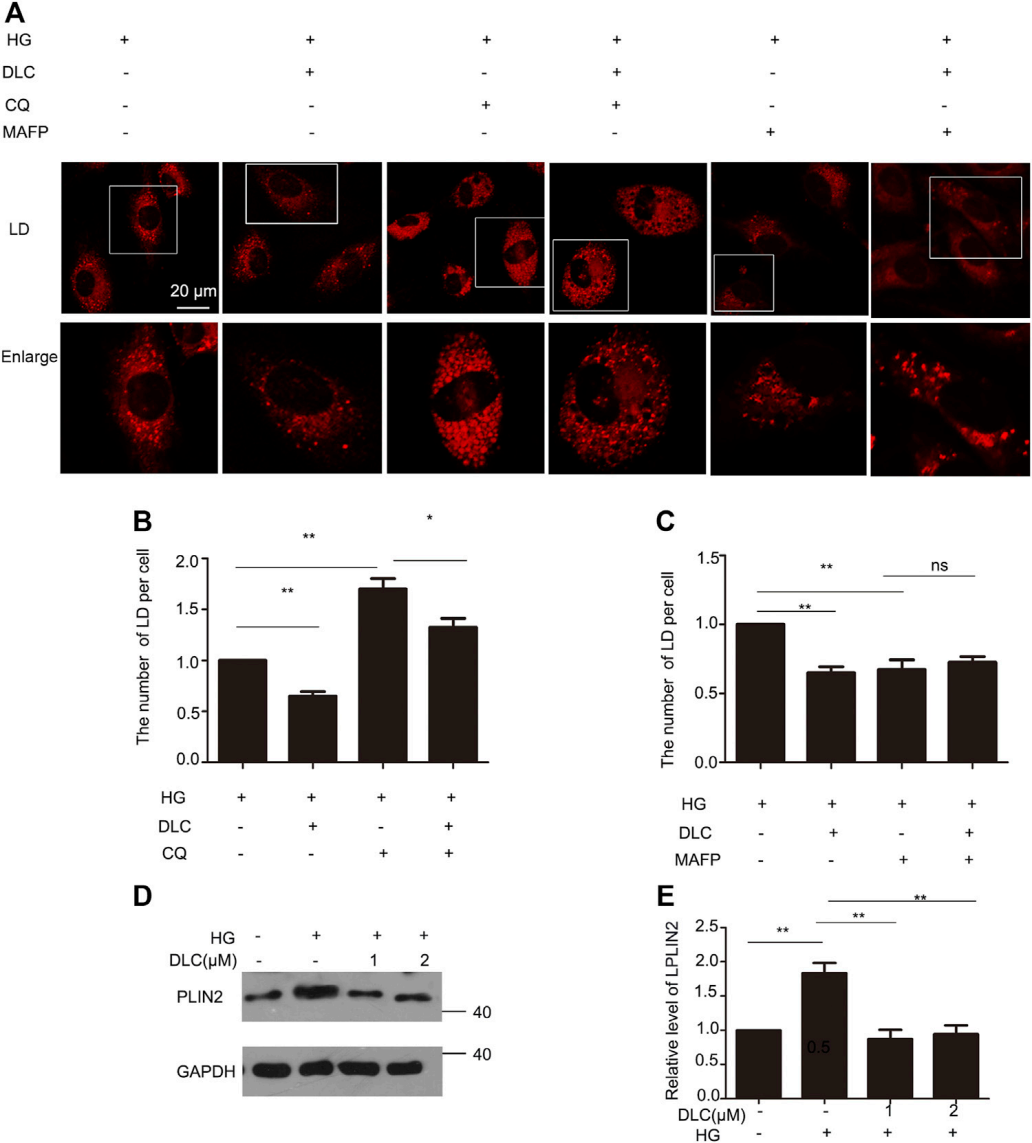
DLC promoted degradation of LDs. **(A,B)** HUVECs were treated with or without MAFP (1 μM) and DLC (1 μM) for 24 h. LDs were stained with neutral lipid stain and counted by ImageJ. **(C,D)** HUVECs were treated with or without CQ (20 μM) and DLC (1 μM) for 24 h. LDs were stained with neutral lipid stain and counted by ImageJ. Scale bar, 20 μm. Data are mean ± SEM; **(D,E)**: HUVECs were treated with DLC (1, 2 μM) under HG condition (30 mM) for 24 h and protein levels of PLIN2 was determined by Western blot. Scale bar: 20 μm, *p < 0.05, **p < 0.01, *n* = 3.

The number of LDs is related to its formation and degradation. Then we used autophagy inhibitor chloroquine (CQ) to inhibit lipid droplet degradation in HUVECs. DLC decreased LDs number under the HG (30 mM) condition (Figures 4A, B). Therefore, DLC promoted degradation of LDs. Next, we used methyl arachidonyl fluorophosphonate (MAFP) to inhibit lipid droplet formation in HUVECs. DLC did not increase LDs number in the presence of MAFP (Figures 4A, C), revealing that the effect of DLC was not associated with formation of LDs.

Perilipins 2 (PLIN2) is a protein on the surface of LDs. Studies have shown that chaperone-mediated selective autophagy (CMA) selectively degrades lipid droplet proteins PLIN2 and PLIN3 from the surface of LDs, thereby promoting cytoplasmic lipase and autophagy effector proteins to LDs recruitment (Kaushik and Cuervo, 2016). More PLN2 protein protects LDs from degradation. Our results showed that DLC decreased the level of PLIN2 under HG condition (Figures 4D, E). These data suggested that DLC could promote the degradation of LDs.

### 3.5 DLC promoted translocation of LDs to lysosomes

**FIGURE 5:**
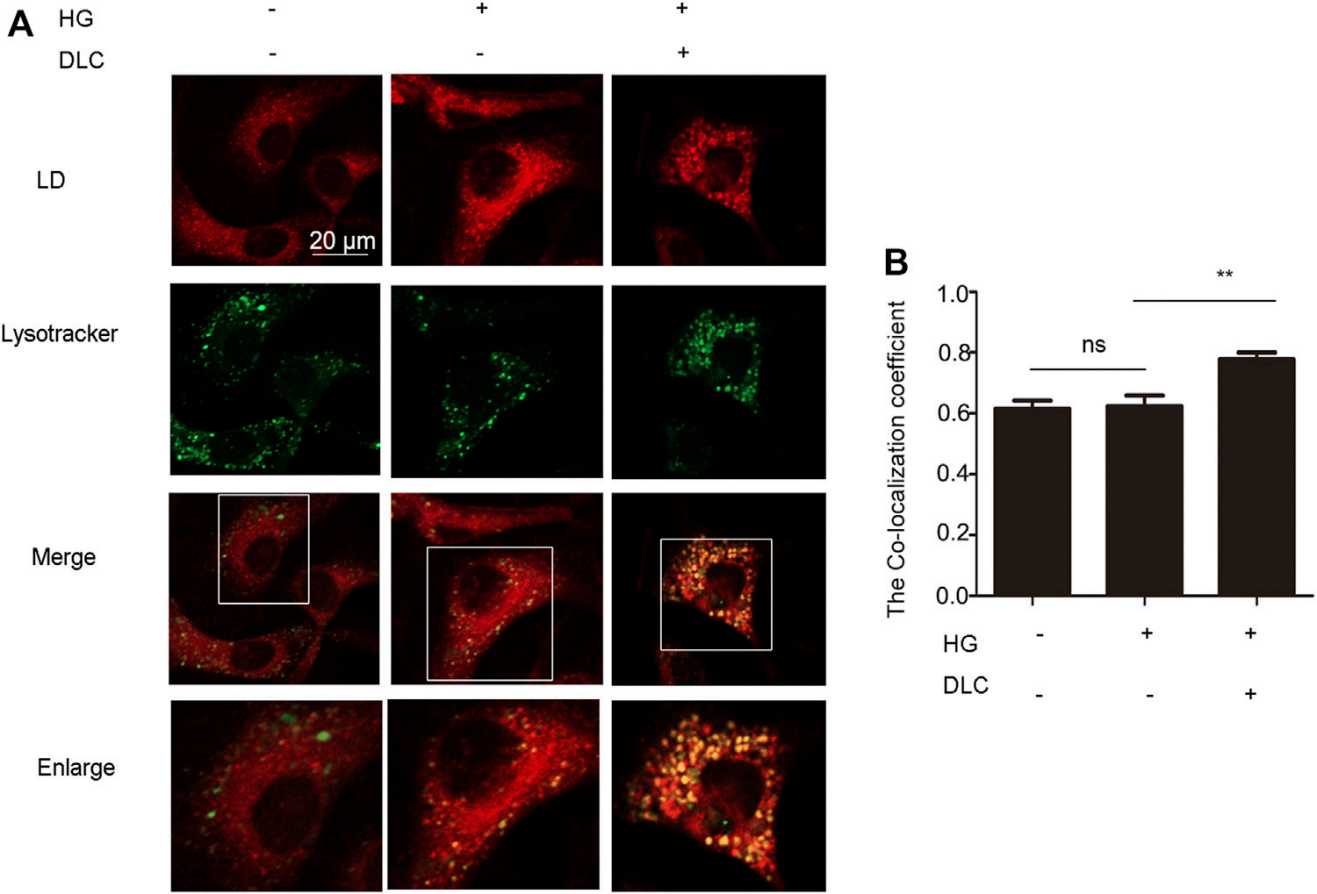
DLC promoted translocation of LD to lysosomes and LDs degradation. **(A,B)**: Co-localization of lysosomes and LDs was detected by immunofluorescence after HUVECs were treated with DLC (1 μM) for 24 h under HG condition (30 mM). Scale bar: 20 μm, **p < 0.01, *n* = 3.

Since LDs reduction is related to the degradation process of LDs, it is reasonable to speculate that lysosomes play an important role in the inhibition of high glucose-induced LDs increase by DLC. Therefore, we examined the effect of DLC on the localization of LDs in cells and found that DLC promoted the co-localization of LDs and lysosomes (Figures 5A, B).

### 3.6 DLC protected LAMP1 level

**FIGURE 6:**
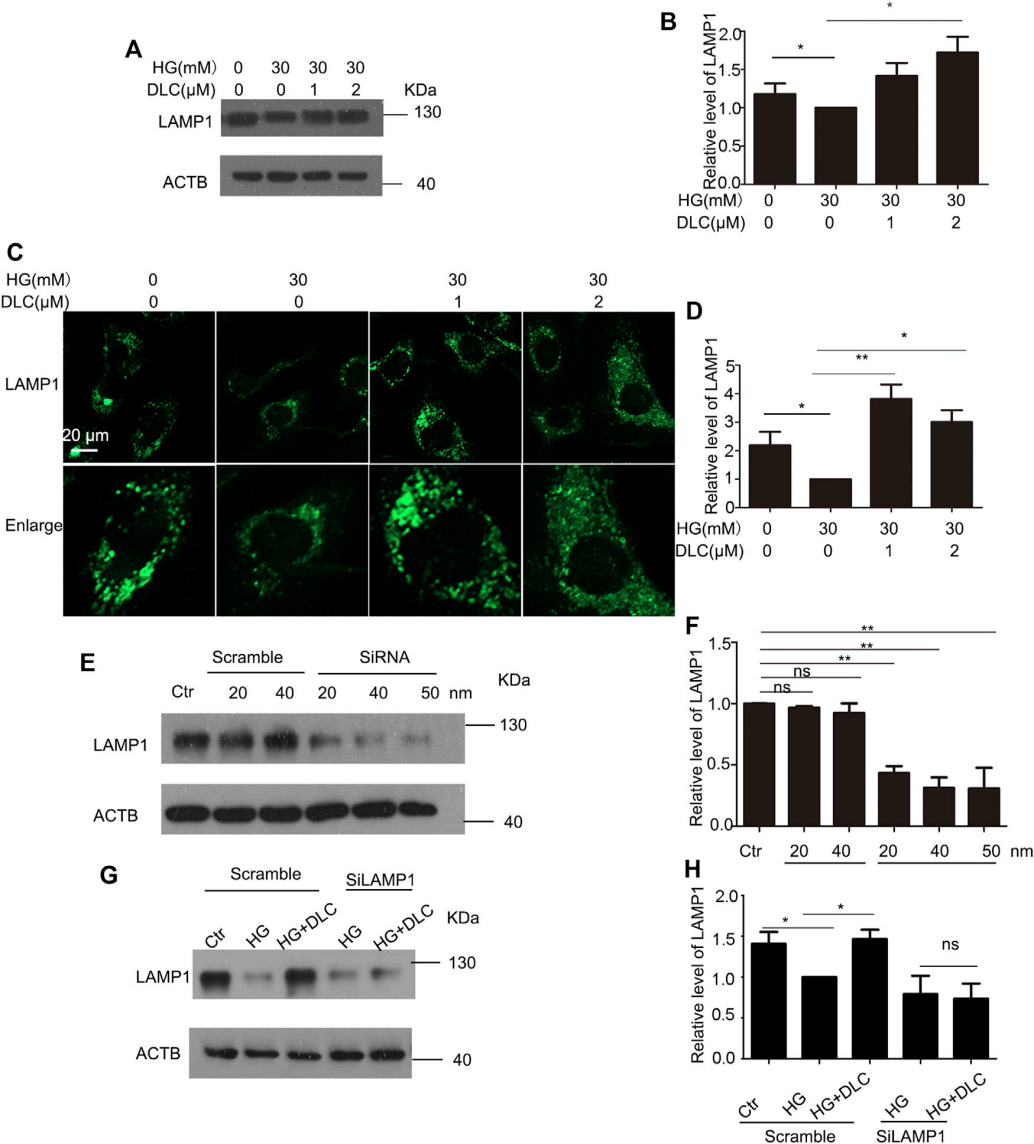
DLC protected LAMP1 level **(A,B)**: HUVECs were treated with DLC (1, 2 μM) for 24 h under HG condition (30 mM) and protein levels of LAMP1 was detected by Western blot. **(C,D)**: HUVECs were treated with DLC (1, 2 μM) for 24 h under HG condition (30 mM) and protein levels of LAMP1 was detected by immunofluorescence **(E,F)**: HUVECs were treated LAMP1 siRNA with under HG condition (30 mM) for 24 h and protein levels of LAMP1 was determined by Western blot. **(G,H)**: HUVECs were transfected with LAMP1 siRNA 24 h, DLC treated HUVECs under HG condition (30 mM) for 24 h and the LAMP1 protein level was detected by western blot. Scale bar: 20 μm, *p < 0.05, **p < 0.01, *n* = 3.

Lysosome-associated membrane protein 1 (LAMP1) is a marker of lysosomal function and protects lysosomal membranes from intracellular proteolysis. Studies have shown that reduced lysosome activity in aging cells leads to lower levels of its membrane protein LAMP1 (Panda et al., 2020). The results showed that DLC could significantly increase the protein level of LAMP1 under HG conditions (Figures 6A, B). To further verify the protective effect of DLC on lysosomal function, we also performed immunofluorescence experiments. Our results showed that DLC could significantly increase the fluorescence level of LAMP1 (Figures 6C, D). In addition, after knocking down LAMP1, DLC no longer increased the level of LAMP1 (Figures 6E–H). These data indicated that DLC could protect lysosomes by increasing the level of LAMP1 and inhibited HG-induced endothelial cell senescence.

### 3.7 DLC protected the proton channel of the V-ATPase

**FIGURE 7:**
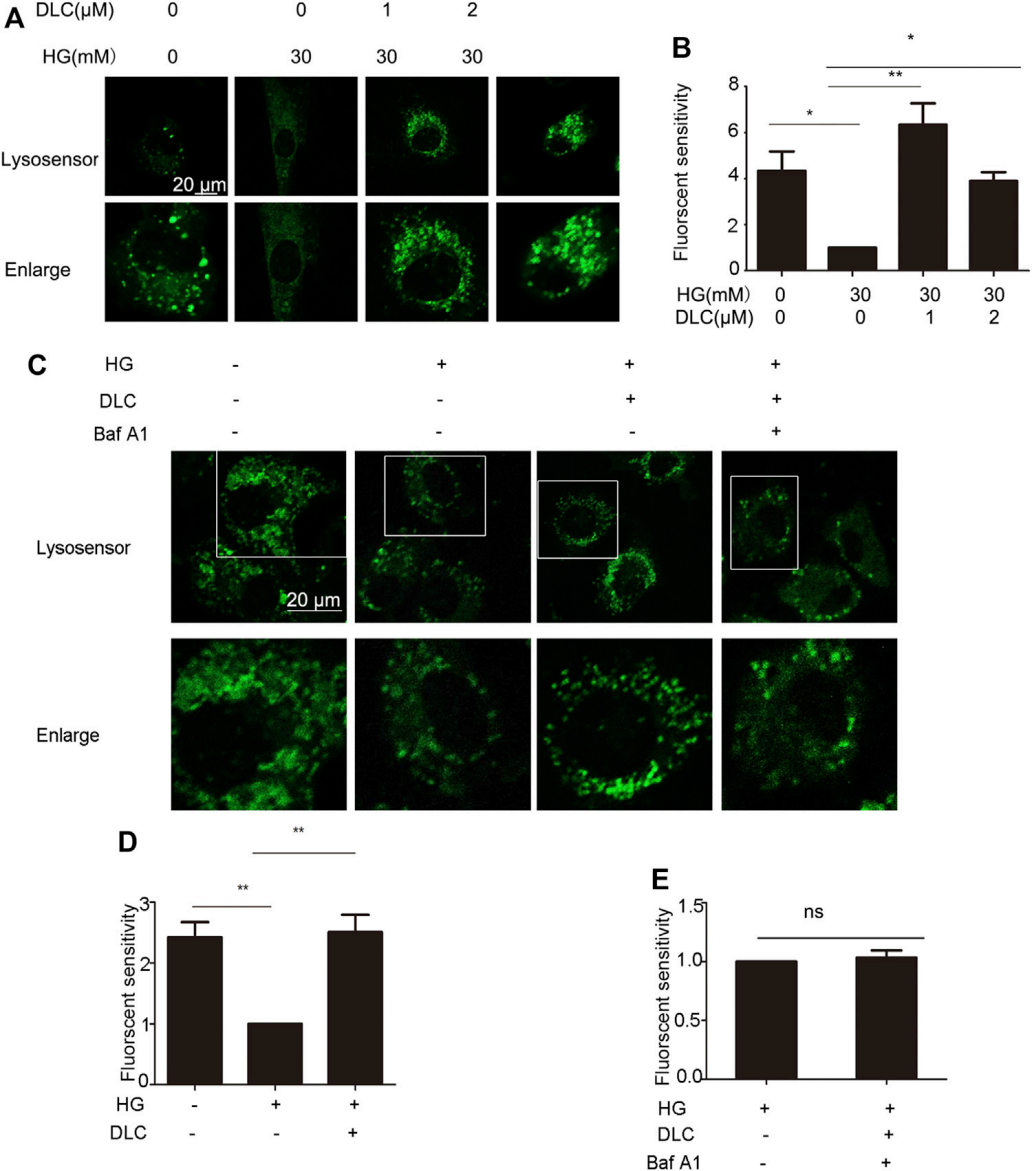
DLC protected the proton channel of the V-ATPase. **(A,B)**: HUVECs were treated with DLC (0, 1, 2 μM) under HG conditionsfor 24 h, H^+^ levels in lysosomes were detected by Lysosensor™ Green DND-189. **(C–E)**: HUVECs were treated with DLC (1 μM) and Baf A1 (5 μM) for 24 h under HG condition, H^+^ levels in lysosomes were detected by Lysosensor™ Green DND-189. Scale bar: 20 μm, *p < 0.05, **p < 0.01, *n* = 3.

Studies have shown that loss of lysosomal acidity leads to lysosomal dysfunction leading to cellular senescence (Nixon, 2020). To investigate whether DLC plays a role by increasing the concentration of H in the senescent lysosome, we incubated the HUVECs with Lysosensor™ Green DND-189, which is used to quantify the H^+^ concentration in the lysosome. The fluorescent intensity results indicated that DLC could significantly increase the concentration of H in lysosome in HUVECs under HG conditions (Figures 7A, B).

The role of V-ATPase is responsible for pumping protons (H) into lysosomes, thereby maintaining the acidic environment of lysosomal cavities and the activity of related hydrolases (Mindell, 2012). However, whether the decrease in lysosomal pH is a consequence of protection of V-ATPase has not been elucidated. The Baf-A1 inhibition site localizes to the V0 proton channel (Wang et al., 2018). As expected, the results indicated that when Baf A1 was co-treated with DLC, DLC no longer increased the H^+^ concentration in lysosomes (Figures 7C–E). These data suggested that DLC can protect the proton channel of V-ATPase on the lysosome.

### 3.8 LAMP1 affected the proton channel of V-ATPase

**FIGURE 8:**
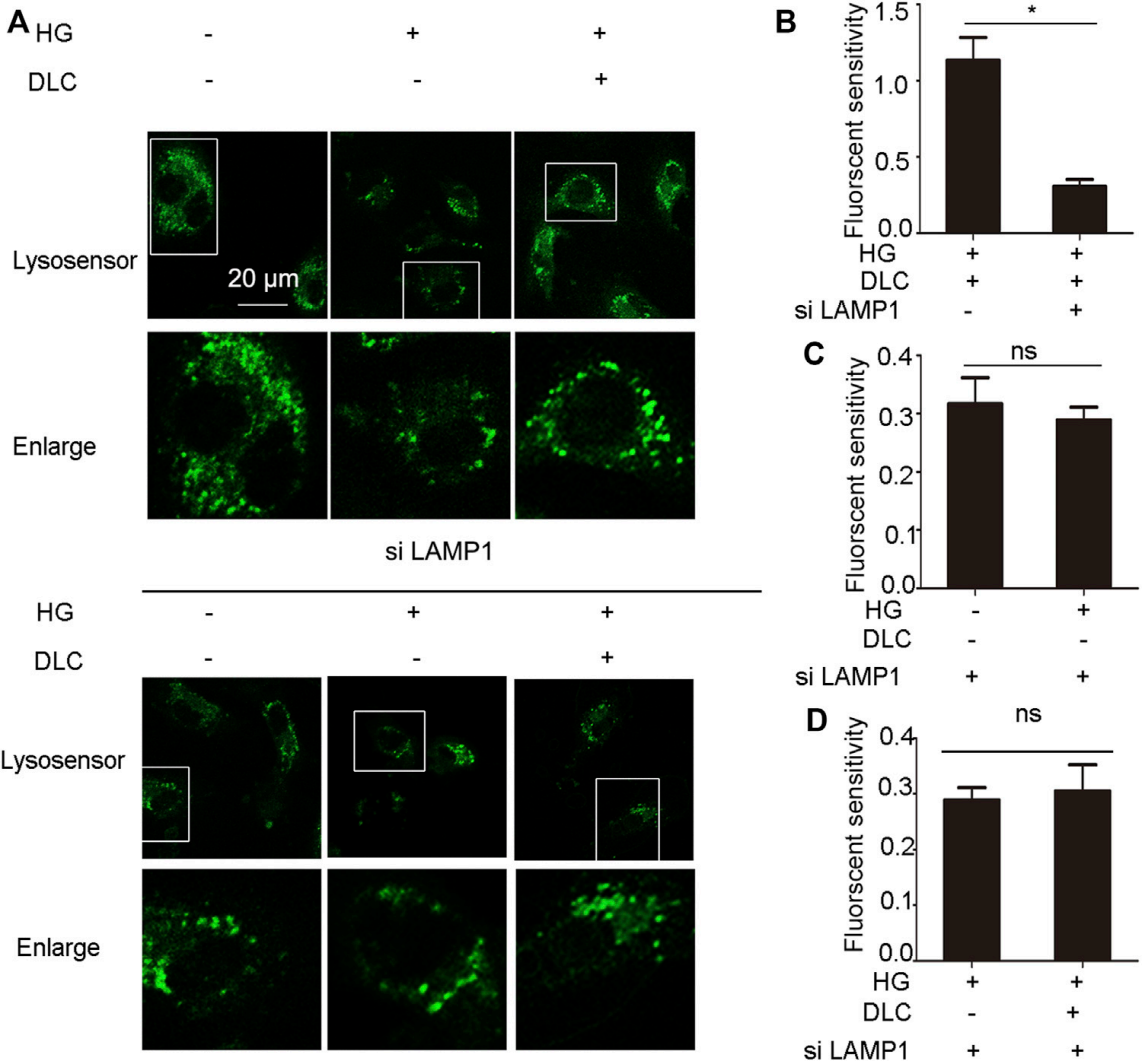
LAMP1 affected the proton channel of V-ATPase. **(A–D)**: HUVECs transmitted LAMP1 SiRNA (40 nM) 24 h, then cells were treated with DLC (1 μM) under HG condition for 24 h, H^+^ levels in lysosomes were detected by Lysosensor™ Green DND-189. Scale bar: 20 μm, *p < 0.05, *n* = 3.

Given that DLC inhibits HG-induced endothelial cell senescence through LAMP1. In addition, DLC can protect the proton channel of V-ATPase on lysosomes, so we speculated that LAMP1 is related to V-ATPase. We found that knockdown of LAMP1 significantly reduced the protective effect of DLC on the proton channel of V-ATPase (Figures 8A, B). In addition, the results further showed that after disrupting LAMP1, DLC no longer increased H^+^ levels in lysosomes compared with the HG group (Figures 8A, C–D). These data indicated that LAMP1 is essential for the proton channel of V-ATPase on lysosome and LAMP1 could regulate the proton channel of V-ATPase.

## 4 Discussion

Studies have shown that high glucose damage to endothelial cells has gradually become the cause of cardiovascular disease (Minamino et al., 2004). Although many studies have revealed various cardiovascular protective effects of SO_2_, its underlying mechanism on HG-induced vascular endothelial cell senescence remains to be determined. In the previous study we synthesized and identified a new ratiometric sulfur dioxide probe (DLC), which has good selectivity for SO_2_ by fluorescence analysis (Yan et al., 2020). However, it is not clear whether SO_2_, especially SO_2_ in the LDs, is involved in the regulation of cell senescence. In this study, we first found that DLC, a SO_2_ probe targeting LDs, inhibited senescence by promoting the degradation of LDs. This provides new evidence for the mechanism of SO_2_ regulating senescence in HUVECs.

Although SO_2_ has been demonstrated to inhibit aging endothelial dysfunction, it remained unclear if LDs are involved in SO_2_ -triggered protection. The dynamics of LDs are tightly regulated by their formation and degradation. Emerging evidence has pinpointed that the generation of LDs is believed to occur in the ER (Kuo et al., 2017). At present, most of research on LD formation has focused on LD formation-related enzymes, including cholesterol O-acyltransferases (ACAT1 and ACAT2) and diacylglycerol acyltrans-ferases (DGAT1 and DGAT2), reside on the ER. Studies have shown that yeast cells lacking these four enzymes are devoid of LDs (Olzmann and Carvalho, 2019). However, Phospholipase A2 enzymes have recently emerged as key regulators of lipid droplet formation (Guijas et al., 2014). In senescent cells, free polyunsaturated fatty acid (PUFA) levels increase *via* p38-dependent activation of phospholipase A2, which cleaves PUFAs from cellular membranes. This allows higher levels of PUFA to be incorporated into triglycerides and eventually accumulate into lipid droplets (Wiley et al., 2019). In this review, we found when MAFP was used to inhibit the formation of LDs at the phospholipase A2 site that DLC did not increase the number of LDs. This implied that DLC is not related to the formation of lipid droplets. However, an understanding of LD formation is incomplete, and many questions remain. For example, we only used inhibitors to inhibit the activity of lipid droplet-related enzymes, but there are many important proteins, such as seipin, involved in the formation of lipid droplets, that we will explore in depth.

The hydrolysis of LDs is mainly regulated through the autophagy-mediated lipolysis, whereby LDs are enclosed within autophagosomal membranes and fused with lysosomes for degradation (Petan et al., 2018). However, it is unclear whether sulfur dioxide is involved in the degradation of LDs by lysosomes. Here, we found that sulfur dioxide probe DLC could decrease the number of LDs and promote translocation of LDs to lysosomes, which provided a new entry point to study the lipolysis of HG-induced senescence. Recent data has implicated that PLIN2 may be selectively recognized by CMA system for LDs degradation. Results indicated that the levels of CMA are elevated in the senescent cells, DLC may alleviate HG-induced senescence by promoting lipolysis.

Lysosomes are the “recycle bins” of cellular catabolism and play important roles in cellular homeostasis, development and aging (Bhat and Li, 2021). Furthermore, There is growing evidence that aging induces the accumulation of LDs in yeast and mammalian cells (Geltinger et al., 2020). In addition, the lysosomal activity and the protein levels of LAMP1 decreased in the process of cell senescence (Wohlgemuth et al., 2007). However, it was unclear whether DLC regulated HUVECs senescence by affecting lysosomal function. Our data demonstrated that DLC promoted the degradation of LDs and promoted the elevation of LAMP1, showing that DLC protected the lysosomal integrity and function to inhibit cellular senescence.

On the lysosomal membrane, the main protein that maintains pH in the acidic lumen is V-ATPase, which is responsible for pumping protons (H) into the lysosome and protect lysosome function (Williamson and Hiesinger, 2010). Recent studies have indicated that increased lysosomal permeability leads to decreased acidity in senescent cells (Han et al., 2020). Interestingly, we found that DLC protected lysosomes by increasing their acidity and LAMP1 regulated the V-ATPase proton channel, which was not reported before. However, the mechanism by which LAMP1 regulates V-ATPase proton channel should be investigated further. Therefore, DLC protected V-ATPase and modulated the acidity of lysosomes as a promising pathway to inhibit aging-related diseases.

**FIGURE 9:**
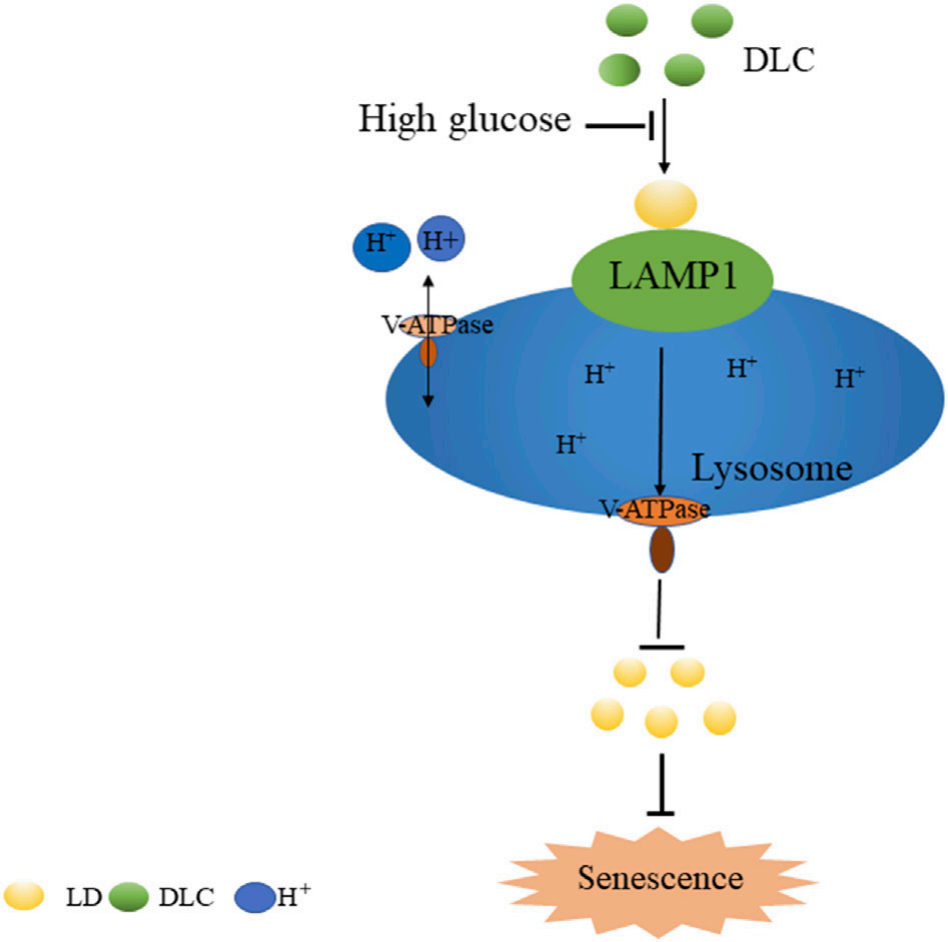
The mechanism of DLC in inhibiting high glucose-induced endothelial cell senescence.

In summary, we screened and identified a sulfur dioxide probe DLC targeted LDs. Our study demonstrated that DLC promoted the level of LAMP1 and protected the V-ATPase proton channel, increased the H^+^ concentration of the lysosome to protect the function of the lysosome. Finally, it promoted the breakdown of LDs to alleviate HG-induced HUVECs senescence (Figure 9), thereby providing new experimental evidence and tools for elucidating the regulatory mechanism of intracellular gas signaling molecule sulfur dioxide on vascular endothelial fate.

## Data Availability

The original contributions presented in the study are included in the article/Supplementary Material, further inquiries can be directed to the corresponding authors.
