# Supplementary material for: A novel sulfur dioxide probe inhibits high glucose-induced endothelial cell senescence
Source: Front Physiol. 2022 Dec 16;13:979986. doi: 10.3389/fphys.2022.979986 (PMC9800602; doi:10.3389/fphys.2022.979986)
Supplement: Supplementary file 1 [file Table1.DOCX]

https://www.jianguoyun.com/p/DU7ITrwQnpzhChio4soEIAA
